# Searching for Lyme borreliosis in Australia: results of a canine sentinel study

**DOI:** 10.1186/s13071-017-2058-z

**Published:** 2017-03-13

**Authors:** Peter J. Irwin, Ian D. Robertson, Mark E. Westman, Martine Perkins, Reinhard K. Straubinger

**Affiliations:** 1 Vector and Water-Borne Pathogen Research Group, School of Veterinary and Life Sciences, Murdoch University, Murdoch, Western Australia 6150 Australia; 2 College of Veterinary Medicine, School of Veterinary and Life Sciences, Murdoch University, Murdoch, Western Australia 6150 Australia; 3 Sydney School of Veterinary Science, University of Sydney, Sydney, New South Wales 2006 Australia; 4 Pymble Veterinary Clinic, Philip Mall, Kendall Street, West Pymble, New South Wales 2073 Australia; 5 Department of Infectious Diseases and Zoonoses, Bacteriology and Mycology, Ludwig-Maximilians-University Munich, 80539 Munich, Germany

**Keywords:** Lyme borreliosis, *Borrelia burgdorferi* (*s.l*.), *Anaplasma*, *Ehrlichia*, *Ixodes*, Ticks, Vector-borne disease, Serology, Canine sentinel, Australia

## Abstract

**Background:**

Lyme borreliosis is a common tick-borne disease of the northern hemisphere that is caused by bacterial spirochaetes of the *Borrelia burgdorferi* (*sensu lato*) (*Bbsl*) complex. To date, there has been no convincing evidence for locally-acquired Lyme borreliosis on the Australian continent and there is currently a national debate concerning the nature and distributions of zoonotic tick-transmitted infectious disease in Australia. In studies conducted in Europe and the United States, dogs have been used as sentinels for tick-associated illness in people since they readily contact ticks that may harbour zoonotic pathogens. Applying this principle, we used a combination of serological assays to test dogs living in tick ‘hot spots’ and exposed to the Australian paralysis tick, *Ixodes holocyclus*, for evidence of exposure to *B. burgdorferi* (*s.l*.) antigens and other vector-borne pathogens.

**Results:**

Altogether, 555 dogs from four demographic groups were recruited into this study. One dog had evidence of exposure to *Anaplasma* spp. but no other dog was positive in screening tests. A total of 122 dogs (22.0%) had a kinetic ELISA (KELA) unit value > 100, and one dog with a high titre (399.9 KELA units) had been vaccinated against *B. burgdorferi* (*sensu stricto*) before travelling to Australia. Older dogs and those with a history of tick paralysis were significantly more likely to have a KELA unit value > 100. Line immunoassay analysis revealed moderate-to-weak (equivocal) bands in 27 (4.9%) dogs.

**Conclusions:**

Except for a single dog presumed to have been exposed to *Anaplasma platys*, infection with *Anaplasma* spp. *B. burgdorferi* (*s.l*.), *Ehrlichia* spp., and *Dirofilaria immitis*, was not detected in the cohort of Australian dogs evaluated in this study. These results provide further evidence that Lyme borreliosis does not exist in Australia but that cross-reacting antibodies (false positive results) are common and may be caused by the transmission of other tick-associated organisms.

## Background

Animals are often the first to come in contact with microbes, contaminants, and pollutants that can cause illness in people and the development of clinical signs in these species can provide early warning for potential threats to human health. Multiple species of wild and domesticated animals have been utilised as sentinels of environmental hazards, including infectious diseases, and serosurveys of dogs have been widely conducted in North America and Europe as an adjunct to the surveillance of human Lyme borreliosis (LB), commonly termed Lyme disease (LD) [1–5]. Dogs are particularly effective sentinels for vector-borne diseases such as LB since their inquisitive behaviour off lead takes them into the long grass and shrub land where they have the potential to come into contact with questing ticks that harbour pathogens. It has been demonstrated that the prevalence of antibodies to *Borrelia burgdorferi* (*s.l*.) (*Bbsl*), the aetiological agents of LB, in endemic areas is significantly greater in dogs than in people [6]. Seropositivity to the LB agent was 0.4–25% in dogs tested in south eastern and mid-Atlantic regions of the USA [7], 8% in dogs in Maine, USA [2], 1.9–10.3% in Germany [8] and 17–18% amongst pet and hunting dogs in The Netherlands [1]. Serosurveys are, however, not without limitations and despite the high sensitivity and specificity of commercially available diagnostic tests, caution is advised when interpreting results, especially from convenience samples in low prevalence populations. Additionally, infection by *Bbsl* results in a lower incidence of clinical illness in dogs than it does in people [9]. It has been concluded that canine seroprevalence to *Bbsl* greater than 5% was a sensitive but non-specific marker of human risk, whereas seroprevalence less than 1% was associated with minimal risk of human infection [10].

In Australia, the diagnosis of LB and a so-called ‘Lyme disease-like syndrome’ has been the subject of much debate, recently resulting in a parliamentary hearing, a Senate enquiry, intense media interest, and three published reviews [11–14]. To date, except for rickettsiosis and coxiellosis, there is no convincing evidence for locally-acquired tick-borne infectious diseases of humans in Australia. Indeed, none of the recognised tick species (the ‘*ricinus*’ complex of *Ixodes*) responsible for vectoring LB and associated pathogens in other parts of the world occurs in Australia, and in one experimental study, it was concluded that Australia does not appear to have a competent vector of *Bbsl* [15]. Current medical opinion regarding positive results of screening antibody tests to *Bbsl*, and other tick-borne pathogens such as *Anaplasma* spp., *Ehrlichia* spp. and *Babesia* spp. in people with no overseas travel is that these most likely represent false positive serological test results [13].

There has been one previous survey for LB-specific antibodies in dogs in Australia, conducted in Brisbane, Queensland over twenty years ago [16]. Although approximately 40% of these dogs had a history of a tick bite, all serum samples were negative. Since that time recombinant purified antigens and peptides derived from the bacteria, including a *Borrelia*-specific lipoprotein VlsE (C6 peptide) have improved the sensitivity of detection of *Bbsl* tests while maintaining specificity in both screening assays and immunoblots. We hypothesised that dogs living within the geographical regions that coincide with the majority of the Lyme disease-like reports in humans (coastal NSW) would offer the greatest probability of detecting antibodies if LB was endemic within the tick populations within those regions. This cross-sectional canine serosurvey was conducted primarily to further the search for evidence of *Bbsl* and other vector-borne infections within Australia, selecting dogs considered most at risk should *Bbsl* be present i.e. targeted serosurveillance.

## Methods

### Collection of samples

**Fig. 1:**
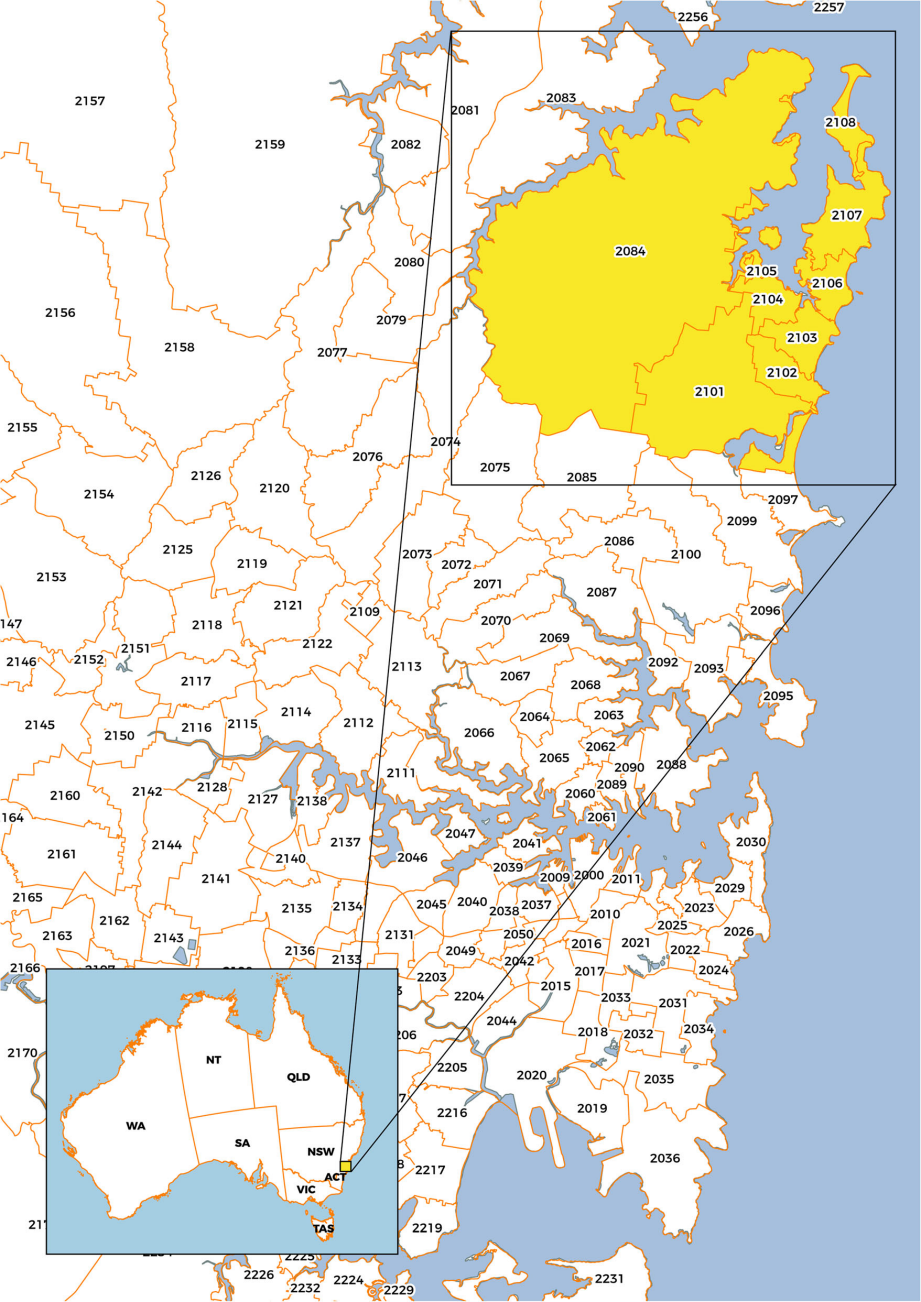
Map showing the Northern Beaches area of Sydney, NSW, postcodes 2101–2108 and 2084, selected for sampling dogs in Group 1. (Map credit: voomMAPS.com)

Between April 2011 and December 2013 dogs were recruited into this cross-sectional study from four sources around Australia as described in Table 1 and Fig. 1. Each dog owner (Groups 1 & 2) completed a questionnaire soliciting information pertaining to the dog’s age, sex, breed, postcode of residence, history of ectoparasite (tick and flea) exposure (including tick paralysis), and travel history (local, interstate and overseas). Additionally, personal medical histories were provided by the owners of dogs in Group 2. Blood samples, collected by veterinarians, were transferred into EDTA-coated and serum (clot) tubes.

**Table 1:** Group details

Group	Number	Description and location
1	381	Dogs (multiple breeds) residing in the Northern Beaches local government area of Sydney, New South Wales (NSW) specifically within postcodes 2101–2108 and 2084 (Fig. 1). This densely populated area of NSW is highly enzootic for the Australian paralysis tick (*Ixodes holocyclus*). Numerous cases of tick paralysis in domestic animals are treated by veterinarians in this area each year and the emergency departments of three local hospitals (Mona Vale, Manly and Hornsby) collectively treated 1,131 tick bite presentations in humans between July 2014 and August 2016 (Dr Ben Taylor, Mona Vale Hospital, 2016 pers. com.). Dogs were recruited by advertisement through local veterinary hospitals and at the Pittwater (now Northern Beaches) Council’s annual event ‘Dog Day by the Bay’ (in 2012 and 2013) at the Rowland Reserve, Bayview. Dogs in this group represented a cohort considered highly likely to be exposed to *I. holocyclus* and therefore act as potential sentinels for human infections.
2	60	Dogs (multiple breeds) owned by and living with people with a variety of symptoms (e.g. headaches, joint and muscle pain, fatigue, sleeplessness, rash, memory loss, etc.) consistent with a ‘Lyme disease-like syndrome’, who had received a diagnosis of a tick-associated illness by a medical practitioner. Owners enrolled their dogs following advertisements by patient advocacy groups and by word of mouth. Dogs assigned to this group were located throughout Australia, but mostly in coastal NSW and Western Australia. Dogs in this group were chosen because of their close association with humans who had received a diagnosis and may, therefore, provide selective evidence for a sentinel status.
3	84	Dogs (foxhounds) resident at Northern Serums Pty Ltd, Lismore NSW, an APVMA-approved manufacturer of paralysis tick antiserum. Most (*n* = 79) of these dogs were bred within the facility, with five adult dogs sourced originally from elsewhere, i.e. Brisbane (*n* = 1) and Melbourne (*n* = 4). At this facility, approximately 400 unfed (questing) female *I. holocyclus* ticks collected from multiple locations in coastal NSW (Lismore North, Casino, Tabulam South, Macksville) and Queensland (Maleny, Atherton and Bauple Mountain) are attached to each dog annually and allowed to feed and engorge before removal. Blood is drawn regularly from these dogs for the manufacture of hyperimmune serum which is supplied commercially to veterinarians for the treatment of tick paralysis in domestic animals. Dogs in this group were chosen to specifically test the hypothesis that dogs bitten by *I. holocyclus* are sentinels for certain infections transmitted by this tick species.
4	30	Dogs (camp dogs; dingo crosses and other breeds) residing at two indigenous communities located on the Dampier Peninsula, north of Broome in the tropical Kimberley region of Western Australia. These dogs were sampled as part of routine health assessments and to determine the internal and external parasite load. In this area, there is a high prevalence of the brown dog tick (*Rhipicephalus sanguineus*) and minimal ectoparasite control. These dogs served as a control group since *I. holocyclus* does not occur in this location.
Total	555	

*Abbreviations*: *APVMA* Australian Pesticides and Veterinary Medicines Authority, *NSW* New South Wales

### Enzyme immunoassay testing

Whole blood was screened for antibodies to *Bbsl*, *Anaplasma phagocytophilum, A. platys*, *Ehrlichia canis* and *E. chaffeensis*, and for *Dirofilaria immitis* antigen, using a rapid enzyme immunoassay (EIA) kit (SNAP® 4Dx and SNAP® 4Dx Plus, IDEXX Laboratories Inc., Westbrook, Maine, USA) according to the manufacturer’s instructions.

### PCR testing

Positive samples from EIA test protocol were sent (on dry ice) for PCR analysis to the Vector Borne Disease Diagnostics Laboratory at North Carolina State University, Raleigh, USA [17].

### ELISA and Line immunoassay

Following centrifugation and separation from blood cells, serum samples were stored at -20 °C at Murdoch University until batched and shipped (on ice) to the Ludwig-Maximilians-University in Munich, Germany, where they were analysed for *Bbsl*-specific antibodies, initially using a computerized kinetic ELISA (KELA) performed as described previously [18]. Briefly, sonicated whole cell-lysate of culture-derived *B. burgdorferi* (*sensu stricto*) N40 served as antigen. Canine sera were diluted 1:100 in PBS with 0.05% of Tween 20 (AppliChem GmbH, Darmstadt, Germany) and 2% milk powder (Merck KGaA, Darmstadt, Germany). Antibodies were detected with HRP-conjugated goat anti-dog IgG (Cappel Laboratories, West Chester, PA, USA) in a dilution of 1:2,000 in PBS with Tween 20 and milk powder after the addition of the TMB substrate system (KPL Inc., Gaithersburg, MD, USA). The developing absorbance of all samples was measured 5 times starting 2 min after the addition of TMB in 35 s intervals at λ = 650 nm with a SpectraMax Plus 384 plate reader (Molecular Devices, LLC., Sunnyvale, CA, USA). The KELA testing was followed by line immunoassay (LIA) to identify the probable targets of the antibodies using the *Borrelia* LIA (Sekisui Virotech GmbH, Rüsselsheim, Germany). Serum samples indicative for dogs which might have had contact with *Borrelia* organisms were defined as those with KELA unit value > 100 [18, 19] and two or more bands in the LIA to the following antigens: VlsE mix, OspA mix (31 kDa), DpbA mix, OspC mix (23 kDa), BmpA (39 kDa), p58, p83/100 [19].

### Statistical analysis

Data were analysed using SPSS version 21. Categorical data were analysed with a Chi-square test for independence and odds ratios (OR) and their 95% confidence intervals (CI) calculated. For the continuous variable (age), an ANOVA was used to compare dogs with KELA units > 100 (equivocal to positive) and those < 100 (negative) after testing for homogeneity of variances and normality. An association between seropositivity and age, sex, ectoparasite exposure, history of tick paralysis and travel was evaluated with *P* < 0.050 considered significant.

## Results

A total of 555 dogs were recruited into this study (Table 1). During initial screening by EIA, a single dog in Group 1 (a 5-year-old male Labrador living in Ingleside, NSW, 2101) returned a positive result to *Anaplasma* spp. antibodies. This dog had a history of tick attachment and tick paralysis, despite the owner reporting use of ectoparasiticides, and had not travelled away from home. Further analysis of a blood sample from this dog was negative on PCR for *Anaplasma* spp. DNA (data not shown). All other dogs tested were negative for *Bbsl*, *Anaplasma* spp. and *Ehrlichia* spp. antibodies, and for *D. immitis* antigen (Table 2).

**Table 2:** Enzyme immunoassay serology test results

Organism tested	Group 1		Group 2		Group 3		Group 4	
	Pos	Neg	Pos	Neg	Pos	Neg	Pos	Neg
*Anaplasma platys/phagocytophilum*	1	380	0	60	0	84	0	30
*Ehrlichia canis/chaffeensis*	0	381	0	60	0	84	0	30
*B. burgdorferi* (*s.l*.) (*Bbsl*) (C6)	0	381	0	60	0	84	0	30
*Dirofilaria immitis*	0	381	0	60	0	84	0	30

*Abbreviations*: *Neg* negative, *Pos* positive

A total of 123 dogs (22.2%) were positive by ELISA (KELA units > 100; Table 3). One dog with a strong positive serological test result (KELA units = 399.4) had lived in the USA and information provided by the owner indicated this individual had been vaccinated against *B. burgdorferi* (*s.s*.) before travelling to Australia. As it was considered this titre was induced by vaccination, data from this dog were removed prior to further statistical analysis.

**Table 3:** Kinetic ELISA serology test results

KELA value (units)	Number	Interpretation
0–99.9	431	negative
100–199.9	118	positive
200–299.9	4	
> 300	1	

There was no sex predilection for antibodies with 24% of male dogs compared with 18% of females positive (*P* = 0.099) (Table 4). Slightly more dogs with a history of previous tick attachment (23.1%) were positive than those without a history of tick attachment (20.4%) and dogs that were positive were significantly older (6.5 years) than negative dogs (5.4 years) (*F*_(1,513)_ = 6.7, *P* = 0.010). A higher percentage of dogs with a history of tick paralysis, implying prolonged attachment of *Ixodes holocyclus*, were positive (29.5%) compared with dogs without a history of tick paralysis (17.4%) (*P* = 0.002). The odds of positivity in dogs with tick paralysis was twice that of dogs without this specific history (Table 4). Neither a history of ectoparasiticide application (*χ* ^*2*^ = 0.871, *df* = 1, *P* = 0.351) nor previous exposure to fleas (*χ* ^*2*^ = 0.009, *df* = 1, *P* = 0.926) was significantly associated with seropositivity. Additionally, there was no significant association between the *Borrelia*-specific antibody levels (KELA units) and location as assessed by postcode (*χ* ^*2*^ = 1.186, *df* = 8, *P* = 0.997). A slightly higher percentage of dogs with a history of travel were seropositive (21.9%) compared with dogs without a history of travel (14.8%) (*P* = 0.064); the odds of positivity in travelling dogs was 1.6 (95% CI: 1.0–2.7) of dogs not travelling (Table 4).

**Table 4:** Details of KELA serology test results with signalment and historical information, and odd ratios

Group	Seropositive KELA units > 100 (*n*)^a^	Seronegative KELA units < 100 (*n*)^a^	Percent positive (95% CI)	OR (95% CI)
1	70	310	18.4 (14.7–22.7)	1.0 (0.5–2.0)
2	11	49	18.3 (9.5–30.4)	1.0
3	32	52	38.1 (27.7–49.3)	2.7 (1.3–6.0)
4	9	21	30.0 (14.7–49.4)	1.9 (0.7–5.3)
Sex				
Male	67	212	24.0 (19.1–29.5)	1.4 (0.9–2.2)
Female	42	191	18.0 (13.3–23.6)	1.0
Tick history				
Yes	91	303	23.1 (19.0–27.6)	1.2 (0.7–2.0)
No	20	78	20.4 (12.9–29.7)	1.0
Tick paralysis				
Yes	61	146	29.5 (23.4–36.2)	2.0 (1.3–3.1)
No	49	233	17.4 (13.1–22.3)	1.0
Flea history				
Yes	46	198	18.9 (14.1–24.3)	1.0 (0.6–1.8)
No	24	106	18.5 (12.2–26.2)	1.0
Ectoparasiticide use				
Yes	57	242	19.1 (14.8–24.0)	1.4 (0.7–2.9)
No	10	60	14.3 (7.1–24.7)	1.0
Travel history				
Yes	50	178	21.9 (16.7–27.9)	1.6 (1.0–2.7)
Stayed at home	28	161	14.8 (10.1–20.7)	1.0
Total	122^b^	432	22.0 (18.6–25.7)	

^a^Data not available from every individual (some incomplete survey responses)

^b^One data point (vaccinated dog) has been removed from this analysis

There was a significant difference in seropositivity between the four groups (*χ* ^*2*^ = 17.094, *df* = 3, *P* = 0.001); the proportion of seropositive dogs using the ELISA was greatest in the group with the highest exposure to *I. holocyclus* ticks (Group 3 dogs used for antiserum production), with dogs in this group 2.7 times more likely to be positive (95% CI: 1.3–6.0) than dogs in Group 2 (Table 4).

Line immunoassay analysis revealed strong bands to recombinant OspA and DpbA antigens in the single vaccinated dog referred to above, and moderate-to-weak (equivocal) bands in a small number (*n* = 29; 4.9%) of other dogs. Three individuals (including the vaccinated dog) had three bands, one dog had two bands, and 25 dogs had a single positive band on LIA.

## Discussion

Canines have been identified in overseas studies to be useful sentinels for tick-transmitted zoonotic disease [20]. The main purpose of this study was to search for evidence of *B. burgdorferi* (*s.l*.), the causative agents of LB, and our results strongly suggest that these pathogens are not present in Australia. We hypothesised that if *B. burgdorferi* (*s.l*.), other related *Borrelia* species, or other zoonotic tick-associated pathogens were present in Australia, dogs exposed to vector ticks would develop antibodies that would be detected by one or more of the serological methods used in this study. Given that the identity of an Australian vector, if present, is unknown, we reasoned that for a locally transmitted tick-associated zoonotic infectious disease, or group of diseases, to become established in Australia, the tick(s) responsible would be relatively widely distributed and well known to attach to and feed on humans. Dogs with increased risk of exposure to *I. holocyclus* were therefore targeted, since this species parasitizes multiple vertebrate hosts, including humans, it belongs to the genus (*Ixodes*) which in the northern hemisphere is responsible for the transmission of LB-causing *Borrelia* organisms, *Anaplasma* spp. and *Babesia* spp. [21], and the geographical distribution of *I. holocyclus* appears to largely coincide with that of the LD-like cases reported in the scientific literature in Australia [12]. Although there are another 18 species of *Ixodes* tick species described in Australia [22], all are confined to the Australian continent; most of these have highly restricted host ranges and/or enzootic distributions, and rarely bite humans [22]. Certainly, if *I. holocyclus* was responsible for the transmission of *Bbsl* to animals or people in Australia, unequivocally positive results would have been detected in the foxhounds comprising Group 3; it is estimated that at the time of sampling these dogs had collectively been hosts to approximately 160,000 female paralysis ticks and that these ticks were representative of multiple locations throughout the species’ enzootic range along the eastern seaboard of Australia.

Using three different serological methods, only one dog in the total cohort of 555 was assessed to have a reliably positive antibody response, and this dog, an 8-year-old female Labrador, was born in the USA, vaccinated against LB as part of a routine vaccination program in the USA, and travelled to Sydney, Australia in 2009, two years prior to being sampled for this study. Antibody levels induced by vaccination start to wane considerably within a few weeks after immunisation but may be detected for years [23]. This dog had the highest KELA value by a considerable margin (>150 KELA units) and three positive bands (very strong positive to OspA, with additional positive bands to DbpA-mix and a 58 kDa recombinant antigen) on LIA. The outer surface protein A (OspA) is a component of all approved LB vaccines. Another outer protein membrane protein, variable major protein-like sequence, expressed (VlsE) contains antigenically variable and invariable regions. Detection of antibody to the sixth invariable region of the VlsE protein (a peptide known as IR6 or the shorter synthetic version C6) has become a reliable serological marker for the diagnosis of LB and is incorporated into the rapid EIA used in this study. However, genes for the C6 peptide are only expressed during replication of *Bbsl* bacteria in the mammalian host, and this peptide is not incorporated into LB vaccines [24]. Unsurprisingly, therefore, the vaccinated dog described above was negative to the C6 antigen, and this result further indicated that no natural exposure to *Bbsl* had occurred in this individual.

Our data indicate that the prevalence of vector-borne infections, as determined by serological responses to a panel of antigens, was very low in the study groups. To some extent this result is unsurprising, since only *A. platys*, *Babesia vogeli* and canine haemoplasmas are reported to be transmitted to dogs by ticks in Australia, and each of these is vectored by the brown dog tick (*R. sanguineus*) [25]. Except for dogs in Group 4, the majority of individuals tested in the current study lived in southern and south-eastern regions of Australia where *R. sanguineus* is relatively uncommon; dogs in these temperate areas are much more likely to be bitten by *I. holocyclus* or the cattle tick *Haemaphysalis longicornis*, neither of which is known to vector the pathogens mentioned above [26]. Furthermore, Australia is currently considered by veterinary authorities to be free from *A. phagocytophilum*, *E. canis* (and *E. chaffeensis*), and members of the *B. burgdorferi* (*s.l*.) complex [27]. The single positive result to *Anaplasma* spp. with the rapid EIA testing could have represented an antibody response to *A. platys*, *A. phagocytophilum*, or a false positive result. One likely explanation that despite living in Sydney and returning a negative PCR result, this dog was at some previous time been bitten by *R. sanguineus* with the subsequent transmission of *A. platys*. This organism causes canine infectious cyclic thrombocytopenia which in most cases results in only mild illness if any. The owners reported prior tick bite (and tick paralysis) in this dog, but information on the identity of ticks on this dog (other than *I. holocyclus*) was not available. The absence of positive antibody results to *A. platys* in dogs in Group 4 was, however, unexpected since these dogs were regularly bitten by *R. sanguineus*, and the prevalence of this pathogen in rural Indigenous communities has been reported previously to be as high as 32% [28]. The absence of heartworm (*D. immitis*) antigen in any of the dogs tested is interesting and is in line with recent unpublished reports of an overall decrease in prevalence throughout Australia as a result of high uptake of heartworm prophylaxis medication [29].

In contrast to the rapid EIA results, approximately 23% of the dogs tested in this study were weakly positive (between 100 and 299.9 units) using the kinetic ELISA (KELA) with a solid phase antigenic substrate derived from whole, cultured *B. burgdorferi* (*s.s*.) organisms. This ELISA is a sensitive test yet has poor specificity especially for equivocal canine serum samples (100–200 KELA units) [18]. The possible explanations for a positive result in this assay include exposure to the *B. burgdorferi* (*s.l*.) genogroup; exposure to another *Borrelia* species (or group), either introduced or endemic to Australia; or cross-reactivity with antigens from other bacteria of unknown identity. The latter two explanations represent false positive results. As noted above, the absence of any sample testing positive for the C6 antigen strongly mitigates against the exposure to *Bbsl* in the dogs tested and furthermore, the absence of specific band patterns in the LIA results also reduces the likelihood of *Bbsl* exposure in this cohort. We believe another reason must be considered to explain this result.

Other *Borrelia* species are known to exist in Australia (reviewed in [12]). Two species of the genus *Borrelia*, *B. theileri* and *B. anserina* (relapsing fever spirochetes), were introduced to the continent by cattle and poultry, respectively, and their vector ticks, during the establishment and development of the Australian agricultural industry since European settlement in 1788. Despite this, borreliosis in the form of relapsing fever in these domesticated animals is seldom diagnosed in Australia and is of relatively little economic impact. The species neither belong to the *B. burgdorferi* (*s.l*.) (*Bbsl*) complex (responsible for LB) nor (of more relevance with regard to serological testing) do the ticks responsible for their transmission, *R*. (*Boophilus*) *australis* and *Argas persicus*, respectively, bite people or dogs with any great frequency [22]. Questions about the presence of native *Borrelia* species, endemic to the Australian continent and therefore (presumably) maintained in sylvatic life-cycles, are largely unanswered at the present time. Spirochaetes were reported in marsupials and native rodents [30, 31] long before any molecular testing was available to reliably identify them, and the vectors of these organisms (if any) are unknown. Very recently DNA of novel *Borrelia* spp. has been amplified from a single *I. holocyclus* tick and from 39% *Bothriocroton concolor* ticks (*n* = 97) feeding on echidnas (*Tachyglossus aculeatus*), a monotreme, in eastern Australia [32, 33]. Phylogenetic analysis has revealed this *Borrelia* species to exist in its own clade, distinct from the LB, Relapsing Fever and Reptile-associated *Borrelia* clades, and possibly represents a grouping that is unique to Australia. Nothing is known yet about its biology or whether it can be transmitted to other animals, including humans, but this seems unlikely given that *B. concolor* is a specialist tick that feeds only from echidnas [22]. Next generation DNA sequencing of large numbers of *I. holocyclus* removed from a wide variety of hosts has, to date, failed to detect any more individual ticks infected with this organism [32, 34]. It seems unlikely therefore that the positive results in the kinetic ELISA test are due to an as yet unidentified *Borrelia* species in Australia.

It is intriguing that the prevalence of KELA seropositivity was higher in dogs with the greatest tick exposure (Groups 3 and 4) and that there was a significant association between seropositivity and tick paralysis, with foxhounds (Group 3) 2.7 times more likely to be seropositive than the dogs in Groups 1 and 2, suggesting a strong relationship between seropositivity and a clinically significant association with *I. holocyclus*. The interval between tick attachment and the development of neurological signs seems to be variable between individuals (and was actually absent in the foxhounds due to their tolerance of the venom) but generally develops between 4 and 5 days after attachment [35]. Regardless, it is plausible that infectious organisms, a potential source for cross-reacting antibodies in our ELISA, may be transmitted from the salivary glands (or midgut) at the same time that venom is injected once the tick is attached to the host. The possible identity of these infectious organisms remains unknown at the present time and is a subject that requires urgent investigation due to its potential to mislead diagnosis. One of the most contentious issues pertaining to the current LB debate in Australia pertains to the detection of antibodies against *Borrelia* spp. (and other pathogens including *Anaplasma* spp., *Ehrlichia* spp. and *Babesia* spp., for example) by laboratories testing serum from people who have never travelled outside Australia. We have conducted similar testing protocols in this study, applied to dogs and utilizing antigens and serological tests that have been developed for known pathogens in the northern hemisphere. Our interpretation of our dogs’ serology, based largely on the absence of reactivity to the C6 antigen and the absence of robust bands by line immunoassay, is that (1) the seroreactivity in nearly a quarter of the dogs tested, especially in those with tick exposure, represents cross-reactivity with antigens of as yet unidentified microorganism(s), (2) LB is an inappropriate diagnosis to make, and (3) appropriate diagnostic tools need to be applied. Furthermore, the microorganism(s) responsible for engendering this antibody response do not appear to be confined to *I. holocyclus* since our control group (Group 4), ostensibly included as a group with no possible exposure to paralysis ticks, yet with high tick exposure (to *R. sanguineus*), also returned positive results in 30% individuals.

## Conclusions

We conclude that vector-borne infections with the pathogens tested in this study were extremely uncommon. Except for a single dog presumed to have been exposed to *Anaplasma platys*, infection with *Anaplasma* spp. *Borrelia burgdorferi* (s.l.), *Ehrlichia* spp. and *Dirofilaria immitis*, was not detected. We, therefore, propose that these results provide further evidence that Lyme borreliosis does not exist in Australia but that cross-reacting antibodies (false positive results), as determined in this study by KELA unit value > 100, are common and may be caused by the transmission of other tick-associated organisms.

## Abbreviations

ANOVA: Analysis of variance
CI: Confidence interval
EDTA: Ethylenediaminetetraacetic acid
EIA: Enzyme immunoassay
KELA: Kinetic enzyme-linked immunosorbent assay
LB: Lyme borreliosis
LD: Lyme disease
LIA: Line immunoassay
NSW: New South Wales
OR: Odds ratio
PCR: Polymerase chain reaction
VlsE: *vmp*-like sequence E
